# The effect of a national web course “Help-Brain-Heart” as a supplemental learning tool before CPR training: a cluster randomised trial

**DOI:** 10.1186/s13049-017-0439-0

**Published:** 2017-09-12

**Authors:** Anette Nord, Leif Svensson, Andreas Claesson, Johan Herlitz, Håkan Hult, Susanne Kreitz-Sandberg, Lennart Nilsson

**Affiliations:** 1 Department of Medical and Health Sciences, Linköping University, S-58185 Linköping, Sweden; 2 Department of Medicine, Center for Resuscitation Science, Karolinska Institute, S-11883 Stockholm, Sweden; 3 Prehospen-Center for Prehospital Research, Faculty of Caring Science, Work Life and Social Welfare, University of Borås, S-50190 Borås, Sweden; 4 Department of Clinical Science, Intervention and Technology, Karolinska Institute, S-14186 Stockholm, Sweden; 5 Department of Behavioural Sciences and Learning, Linköping University, S-58183 Linköping, Sweden

**Keywords:** CPR training, Web course, Willingness, Infarction, Stroke, Lifestyle factors, Students

## Abstract

**Background:**

The effectiveness of cardiopulmonary resuscitation (CPR) learning methods is unclear. Our aim was to evaluate whether a web course before CPR training, teaching the importance of recognition of symptoms of stroke and acute myocardial infarction (AMI) and a healthy lifestyle, could influence not only theoretical knowledge but also practical CPR skills or willingness to act in a cardiac arrest situation.

**Methods:**

Classes with 13-year-old students were randomised to CPR training only (control) or a web course plus CPR training (intervention). Data were collected (practical test and a questionnaire) directly after training and at 6 months. CPR skills were evaluated using a modified Cardiff test (12–48 points). Knowledge on stroke symptoms (0–7 points), AMI symptoms (0–9 points) and lifestyle factors (0–6 points), and willingness to act were assessed by the questionnaire. The primary endpoint was CPR skills at 6 months. CPR skills directly after training, willingness to act and theoretical knowledge were secondary endpoints. Training and measurements were performed from December 2013 to October 2014.

**Results:**

Four hundred and thirty-two students were included in the analysis of practical skills and self-reported confidence. The mean score for CPR skills was 34 points after training (control, standard deviation [SD] 4.4; intervention, SD 4.0; not significant [NS]); and 32 points at 6 months for controls (SD 3.9) and 33 points for intervention (SD 4.2; NS). At 6 months, 73% (control) versus 80% (intervention; *P* = 0.05) stated they would do compressions and ventilation if a friend had a cardiac arrest, whereas 31% versus 34% (NS) would perform both if the victim was a stranger. One thousand, two hundred and thirty-two students were included in the analysis of theoretical knowledge; the mean scores at 6 months for the control and intervention groups were 2.8 (SD 1.6) and 3.2 (SD 1.4) points (*P* < 0.001) for stroke symptoms, 2.6 (SD 2.0) and 2.9 (SD 1.9) points (*P* = 0.008) for AMI symptoms and 3.2 (SD 1.2) and 3.4 (SD 1.0) points (*P* < 0.001) for lifestyle factors, respectively.

**Discussion:**

Use of online learning platforms is a fast growing technology that increases the flexibility of learning in terms of location, time and is available before and after practical training.

**Conclusions:**

A web course before CPR training did not influence practical CPR skills or willingness to act, but improved the students’ theoretical knowledge of AMI, stroke and lifestyle factors.

**Electronic supplementary material:**

The online version of this article (10.1186/s13049-017-0439-0) contains supplementary material, which is available to authorized users.

## Background

The Chain of Survival summarises the vital steps needed for neurologically intact survival after out-of-hospital cardiac arrest (OHCA) [1]. The first link, “early recognition and call for help”, includes identification of symptoms of acute myocardial infarction (AMI), identification of cardiac arrest and early activation of emergency medical services. A quarter of cardiac arrest patients have myocardial ischaemic symptoms before cardiac arrest occurs [2]. The second link describes the importance of early cardiopulmonary resuscitation (CPR). Immediate CPR can increase the chance of survival two to four times [1, 3, 4]. The third link is early defibrillation, which can result in survival rates as high as 50–70% [1].

Use of online learning platforms is a fast-growing technology [5]. Sweden is in third place in the world (after Singapore and Finland) for the use of the internet among individuals and companies, with 91% of young people aged 12–15 years using the internet on a mobile phone daily [6, 7]. Reder et al. [8] have shown that an interactive computer session in combination with practical CPR training was an instructive method for high school students. In another study, watching an instructional video before practical infant CPR training improved skill acquisition compared with a traditional training method [9]. In October 2016, the European Resuscitation Council (ERC) started an online learning system, CoSy, in preparation for practical basic life support training [10].

A statement from the ERC, approved by the World Health Organization (WHO), recommends that all school children should undergo CPR training every year starting from the age of 12 years [11]. If all students undergo practical CPR training in school, a large proportion of a nation’s population would have basic skills within a few decades. Such a situation could improve the rate and quality of CPR intervention by bystanders for OHCA and have a significant effect on survival and public health [11–13].

CPR training can be organised in various formats (e.g. instructor-led or self-instruction kits, practical or theoretical, video-based, app-based, e-learning). The effectiveness of different CPR learning methods is unclear [14]. The present study evaluated an interactive web course, Help-Brain-Heart (HBH), teaching symptoms of stroke, symptoms of AMI and a healthy lifestyle [15]. A preparatory web course and information on stroke, AMI or healthy lifestyles factors are not included in traditional basic life support training. The aim was to investigate whether this web course given to 13-year-old students before CPR training could influence not only theoretical knowledge about stroke, AMI and lifestyle factors but also practical CPR skills and willingness to act in a cardiac arrest situation. CPR quality was assessed directly after training and at follow-up 6 months later. Our hypothesis was that basic knowledge of cardiovascular disease, through the web course, places CPR knowledge in context and increases acquisition of practical CPR skills.

## Methods

### Study population and design

Academic leaders of all 24 council schools in two Swedish municipalities (each with 140,000 inhabitants) received an invitation to participate in the study. In accordance with the school curriculum in Sweden [16], the intervention was applied in grade 7 (13-year-old students). Before the study, the students and their parents received a letter about the study. Participation by individual students was completely voluntary and was preceded by oral informed consent.

All seventh grade students in each participating school were eligible to take part. Students were excluded if they declined participation, had a physical handicap that significantly limited their performance, or had development disabilities.

A cluster randomised design was applied in the present study [17]. Based on a randomisation list generated by an independent statistician, classes were cluster randomised to CPR training only (control group) or to a web course before practical CPR training (intervention group). CPR training and data collection took place between December 2013 and October 2014.

### Practical CPR training applied to all groups

Both the control group and the intervention group received the same practical CPR training. All the students had an individual training manikin, MiniAnne (Laerdal AS, Norway), during training. All training sessions were carried out in accordance with the current ERC guidelines [18]. School teachers, who all were CPR instructors, acted as facilitators and were responsible for training the pupils in CPR. All teachers obtained individual oral and written information to ensure that they were up to date with the present CPR guidelines and training. Training was given to the entire class together. The CPR training was based on a mobile application (app) or a video on DVD [19]. The study design and the CPR training have been described elsewhere [19]. The web course, the DVD and the app were all produced by the Swedish CPR Council and based on ERC guidelines 2010 [18].

### Web course: Help-brain-heart

The HBH web course was developed and published online by the Swedish Resuscitation Council in 2013 and deals with the symptoms, causes and actions for stroke, AMI and cardiac arrest and includes information about lifestyle factors (smoking, exercise and diet) [15]. The web course is led by an animated storyteller. The narrative is interspersed with videos, animations and ten interactive questions. The purpose of the interactive questions is to engage the students and force them to use decision-making skills. This web course lasts for 20–30 min. If the students had access to a computer, the web course was conducted individually during a lesson, otherwise by the whole class together. Students in the intervention group undertook the web course within 7 days before the practical CPR training.

### Assessment

Directly after CPR training and at the 6-month follow-up, the students were given a practical CPR skill test and asked to complete a questionnaire. All tests were performed individually and carried out at the schools. As previously described, the Laerdal PC skill reporting system 2.4 linked to ResusciAnne (Laerdal NS, Norway) was used to measure the quantitative data for CPR performance, whereas “check responsiveness”, “check respiration” and “alarm 112” were assessed by direct observation by the investigator [19]. The test data were recorded directly into a modified version of the validated Cardiff test [20], on a scoring sheet adapted to current resuscitation guidelines [18]. A total score was calculated for each student (12–48 points). The indicators on the scoring sheet are described in detail in Additional file 1.

The ERC guidelines recommend a compression depth of 50–60 mm [18]. The PC Skill Reporter System measures compression depth up to 60 mm. To avoid a situation where those who compressed > 60 mm were given the highest score (6 points), the highest score was given for an average compression depth of 50–59 mm. We chose to retain the 6-point scale, as in previous studies [21], even though no one could receive the 3 points corresponding to a compression depth > 65 mm (Additional file 1).

The duration of the practical test was 3 min. The optimal conduct was a maximum of 30 s to check responsiveness, check respiration and call for help, followed by 2.5 min of CPR. As an introduction to the practical test, the test leader described an OHCA situation and asked the student to act accordingly. Directly after the test, the student received individual constructive feedback from the test leader for 2 min. The test at 6 months was carried out in the same way and without prior notice. The test leader (AN) is a certified CPR instructor, experienced in the modified Cardiff test, who was blinded to the training method of the students.

After the practical test, the students answered a fixed-response questionnaire with questions about background factors, self-reported confidence, willingness to act, and knowledge about stroke, AMI and lifestyle factors (Additional file 2). Most students responded to the survey online and each question had to be answered in order to proceed to the next. The survey took 6–15 min to complete. The questionnaire resulted in a total score of 0–7 points for stroke symptoms, 0–9 points for AMI symptoms and 0–6 points on lifestyle factors. A higher score meant better knowledge (correct answers). Questions about AMI were partly based on a previous survey [22]. Before the study, comprehension of the questions about self-efficacy and scenarios about willingness to act were tested and found satisfactory in a separate cohort of 175 students.

### Study outcome measures

The primary endpoint was the total score for the modified Cardiff test at 6 months. The score directly after training, the individual variables in the practical test, self-reported willingness to make a lifesaving intervention and theoretical knowledge about stroke, AMI and lifestyle factors were secondary endpoints.

### Statistical analysis

**Fig. 1:**
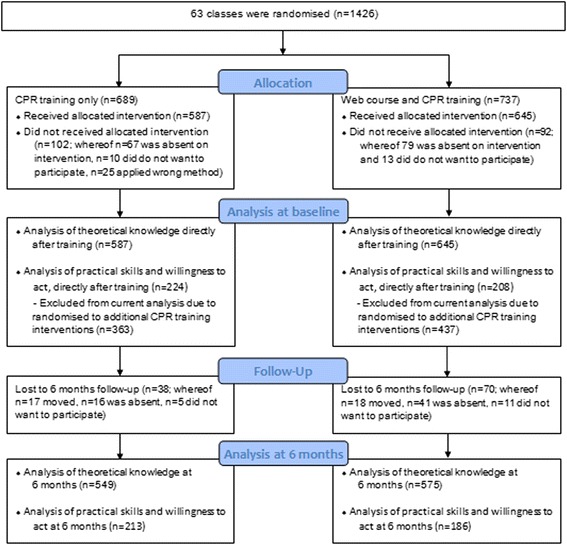
Flowchart for randomisation and inclusion

The results for all participants were included in the analysis of the students’ theoretical knowledge about stroke, AMI and lifestyle factors. For the analysis of the participants’ practical CPR skills and self-reported confidence, some classes were excluded because they were randomised to various additional interventions (automated external defibrillator training, reflection, visit from elite athletes) that may have affected their practical skills or willingness to act and which are reported in previous articles (Fig. 1 **)** [19, 23]. Sample size calculations were based on pre-study data [24]. In order to detect a two-point difference in the total score for the modified Cardiff test, at a significance level of 0.05, an effective sample size of 76 students was needed to test for superiority with a power of 80%. The intraclass correlation coefficient (95% confidence interval) was 0.18 (0.16, 0.21) [17, 25].

Questions on knowledge about stroke, AMI and lifestyle factors were scored as follows: 1 point for a correct answer, 0 points for an incorrect answer and for “do not know”. Data are presented as proportions (percent) or means (standard deviation). Differences in mean total score between the intervention groups were assessed using the independent samples *t* test. To account for a potential cluster effect of the school classes, a mixed models linear test was also applied for comparisons of the total score. A logistic regression within generalised estimation equations was applied for a comparison of categorical variables [17]. By calculating (individual total score − 12)/(maximum total score − 12) × 100, we obtained a measure of CPR quality in relation to optimal CPR. A *P* value < 0.05 was considered significant. Analyses were performed using IBM SPSS version 21 and STATA version 13.1.

## Results

A total of 1426 seventh grade students from 63 classes (each with 14–29 students) in 18 schools were randomised to CPR training only or to receive a web course before CPR training. Directly after training, 1232 students, corresponding to 86% of eligible students, were included in the study, and at 6 months 1124 (91%) of these students completed the follow-up assessment. Four hundred and thirty-two participants were included in the analysis of practical CPR skills and self-reported confidence directly after training and 399 at 6 months (Fig. 1). The characteristics of the students are shown in Table 1.

**Table 1:** Characteristics of the students

	Total	CPR training only	Web course + CPR training	*P* value
Students included in the analysis of the practical test and self-reported confidence	432	224	208	
Male	213 (49)	116 (52)	97 (47)	NS
Previous compression training	124 (29)	54 (24)	70 (34)	NS
Previous ventilation training	96 (22)	41 (18)	55 (26)	NS
App method	205 (48)	113 (50)	92 (44)	NS
DVD method	227 (52)	111 (50)	116 (56)	NS
All students	1232	587	645	
Male	579 (47)	283 (48)	296 (46)	NS
Previously experienced an AMI situation	86 (7)	43 (7)	43 (7)	NS
Previously experienced a stroke situation	58 (5)	25 (4)	33 (5)	NS
Previously experienced a cardiac arrest situation	40 (3)	21 (4)	19 (3)	NS
Previous compression training	363 (30)	152 (26)	211 (33)	NS
Previous ventilation training	271 (22)	114 (19)	157 (24)	NS

Values are presented as *n* (%). Differences in proportions between groups were analysed with logistic regression within generalised estimation eqs. NS, not significant

Based on an average cluster size of 21.6 (the number of clusters was 20), the design effect caused by the cluster randomisation was 4.71. A total of 432 and 399 students performed the first and the second practical CPR test, respectively. This corresponds to an effective sample size of 92 and 85, respectively, which is above the 76 persons needed to reach a power of 80%.

### CPR skills

There was no significant difference between the two groups with regard to total score for the modified Cardiff test. Directly after CPR training, the intervention group scored 34 points (SD 4.0; 61% of the maximum score) and the control group scored 34 points (SD 4.4; 61% of maximum score). At the 6-month follow-up, the groups scored 33 points (SD 4.2; 58% of the maximum score) versus 32 points (SD 3.9; 56% of the maximum score; Table 2).

**Table 2:** Assessment of CPR skills directly after training and at 6 months

	CPR training only, directly after (*n* = 224)	Web course + CPR training, directly after (*n* = 208)	*P* value	CPR training only, after 6 months (*n* = 213)	Web course + CPR training, after 6 months (*n* = 186)	*P* value
Checks responsiveness by talking						
2: Yes	106 (47)	140 (67)	0.011	160 (75)	117 (63)	NS
1: No	118 (53)	68 (33)		53 (25)	69 (37)	
Checks responsiveness by shaking						
3: Yes	147 (66)	154 (74)	NS	154 (72)	115 (62)	NS
2: No	76 (34)	54 (26)		59 (28)	71 (38)	
3: Potentially dangerous	1 (< 1)	0		0	0	
Open airway – chin lift, head tilt						
5: Perfect	4 (2)	6 (3)	Ref	1 (< 1)	3 (2)	ref
4: Acceptable	38 (17)	26 (12)	NS	6 (3)	6 (3)	NS
3: Attempted other	0	0		0	1 (< 1)	
2: Only one element	54 (24)	68 (33)	NS	21 (10)	18 (10)	NS
1: No	128 (57)	108 (52)	NS	185 (87)	158 (85)	NS
Checks respiration - see, listen, feel						
2: Yes	163 (73)	155 (74)	NS	97 (46)	87 (47)	NS
1: No	61 (27)	53 (26)		116 (54)	99 (53)	
Call 112						
2: Yes	161 (72)	166 (80)	NS	171 (80)	149 (80)	NS
1: No	63 (28)	42 (20)		42 (20)	37 (20)	
Compression/ventilation ratio						
4: 30:2 (28–32:2)	95 (42)	94 (45)	Ref	67 (32)	84 (45)	ref
3: Other	117 (52)	102 (49)	NS	129 (61)	88 (47)	NS
2: Compressions only	12 (5)	12 (6)	NS	17 (8)	14 (8)	NS
1: Ventilations only	0	0		0	0	
Hand position during compression						
4: Correct	21 (9)	24 (12)	Ref	7 (3)	9 (5)	ref
3: Other wrong	130 (58)	130 (62)	NS	107 (50)	96 (52)	NS
2: Too low	73 (32)	54 (26)	NS	99 (46)	81 (44)	NS
1: Not attempted	0	0		0	0	
Average compression depth						
6: 50–59 mm	43 (19)	39 (19)	Ref	79 (37)	53 (28)	ref
5: ≥60 mm	1 (< 1)	2 (1)	NS	6 (3)	4 (2)	NS
4: 35–49 mm	113 (50)	110 (53)	NS	97 (46)	86 (46)	NS
2: 1–34 mm	67 (30)	57 (27)	NS	31 (15)	43 (23)	NS
1: Not attempted	0			0	0	
Total compression counted						
6: 140–190	81 (36)	89 (43)	Ref	75 (35)	75 (40)	ref
5: ≥191	107 (48)	92 (44)	NS	100 (47)	87 (47)	NS
4: 121–139	18 (8)	14 (7)	NS	19 (9)	10 (5)	NS
3: 81–120	7 (3)	11 (5)	NS	14 (7)	12 (6)	NS
2: 1–80	11 (5)	2 (1)	0.013	5 (2)	2 (1)	NS
1: Not attempted	0	0		0	0	
Average ventilation volume						
5: 500–600 ml	17 (8)	11 (5)	Ref	7 (3)	11 (6)	ref
4: 1–499 ml	18 (8)	21 (10)	NS	21(10)	20 (11)	NS
3: ≥601 ml	128 (57)	99 (48)	NS	91 (43)	72 (39)	NS
2: 0 ml	49 (22)	65 (31)	NS	77 (36)	69 (37)	NS
1: Not attempted	12 (5)	12 (6)	NS	17 (8)	14 (8)	NS
Total ventilation counted						
5: 8–12	79 (35)	65 (31)	Ref	34 (16)	47 (25)	ref
4: 1–7	52 (23)	43 (21)	NS	44 (21)	31 (17)	NS
3: ≥13	32 (14)	23 (11)	NS	41 (19)	25 (13)	0.05
2: 0	49 (22)	65 (31)	NS	77 (36)	69 (37)	NS
1: Not attempted	12 (5)	12 (6)	NS	17(8)	14 (8)	NS
Total hands-off time						
4: 0–60 s	32 (14)	34 (16)	Ref	62 (29)	66 (36)	ref
3: 61–90 s	147 (66)	122 (59)	NS	120 (56)	99 (53)	NS
2: 91–135 s	39 (17)	50 (24)	NS	30 (14)	21 (11)	NS
1: 136–180 s	6 (3)	2 (1)	NS	1 (< 1)	0	
Total score	34 (4.4)	34 (4.0)	NS^a,b^	32 (3.9)	33 (4.2)	NS^a,b^

Results are presented as n (%) or mean (SD). Differences in proportions between groups were analysed with logistic regression within generalised estimation eqs. *P* values < 0.05 were considered statistically significant. NS, not significant. The table lists the variable’s best option at the top. All numbers are rounded to the nearest integer

^a^Differences in total score between intervention groups were analysed by mixed models linear test

^b^Unpaired t test

### Self-reported confidence and willingness to act

Directly after training, most students responded that they would do both compressions and ventilation if a friend suffered OHCA (80% in both groups; not significant [NS]). If a stranger suffered OHCA, a lower proportion of the students in both the intervention and control groups would perform both compressions and ventilation (42% versus 32%, respectively; NS). Most of the students (98% in both groups; NS) indicated that it is important to learn CPR in school and they felt more confident about acting compared with before the training (82% of the intervention group and 88% of the control group; NS). Also, students considered themselves to have enough knowledge to do chest compressions (82% in the intervention group and 81% in the control group; NS) and to do rescue breaths (78 and 75%, respectively; NS).

At 6 months, 80% (intervention) and 73% (control, *P* = 0.05) of the students responded that they would do both compressions and ventilation if a friend suffered OHCA; there was no significant difference in the situation involving a stranger (34 and 31%, respectively; NS). Again, most of the students considered themselves to have enough knowledge to do chest compressions (93% in the intervention group and 90% in the control group; NS), to do rescue breaths (78 and 73%, respectively; NS) and felt more confident to act compared with before the training (84 and 82%, respectively; NS).

### Theoretical knowledge of stroke, AMI and lifestyle factors

**Fig. 2:**
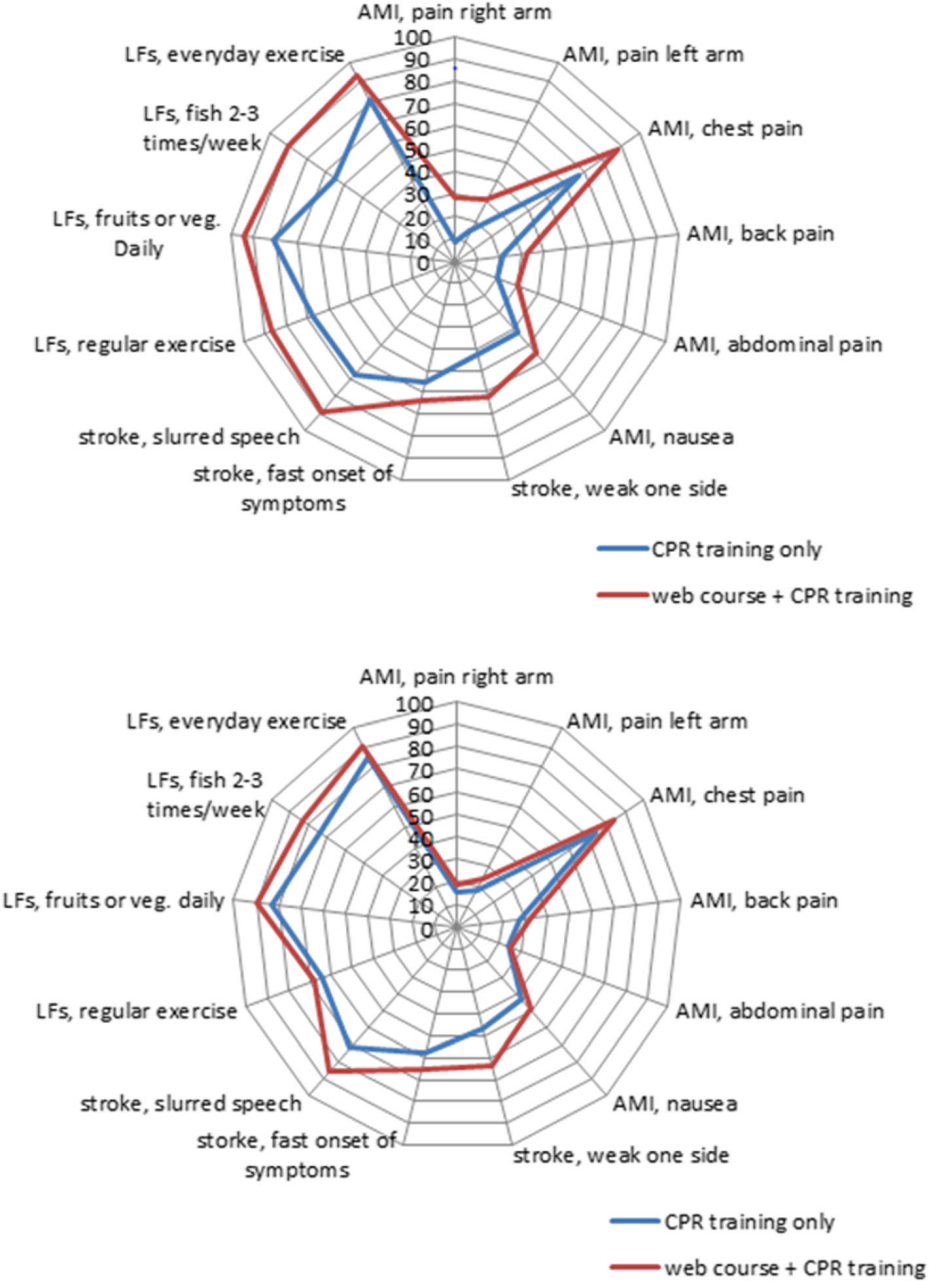
Students’ theoretical knowledge about symptoms of stroke, acute myocardial infarction (AMI) and lifestyle factors (LF). Upper panel: assessment directly after training (CPR training only, *n* = 587; web course + CPR training, *n* = 645). Lower panel: assessed after 6 months (CPR training only, *n* = 549; web course + CPR training, *n* = 575). Values are given as the percentage of correct answers

The intervention group scored significantly better in terms of total score for the survey questions regarding stroke, AMI and lifestyle factors, both directly after training and at the 6 months follow-up (Table 3). Figure 2 shows the students’ knowledge per symptom. Values for all the variables are presented in Additional file 3.

**Table 3:** Total scores for the questionnaire directly after training and after 6 months

	CPR training only, directly after (*n* = 587)	Web course + CPR training, directly after (*n* = 645)	*P* value	CPR training only, after 6 months (*n* = 549)	Web course + CPR training after 6 months (*n* = 575)	*P* value
Stroke symptoms, total score	2.7 (2.0)	3.8 (1.8)	< 0.001^a,b^	2.8 (1.6)	3.2 (1.4)	< 0.001^a,b^
AMI symptoms, total score	2.5 (2.0)	4.0 (2.0)	< 0.001^a,b^	2.6 (2.0)	2.9 (1.9)	0.008^a,b^
Lifestyle factors, total score	4.5 (2.0)	5.4 (1.2)	< 0.001 ^a,b^	3.2 (1.2)	3.4 (1.0)	< 0.001^a,b^

The results are presented as the means (SD). *P* values < 0.05 were considered statistically significant. Theoretical knowledge resulted in a total score of 0–7 points for stroke symptoms, 0–9 points for AMI symptoms and 0–6 points on lifestyle factors

^a^Differences in total score between intervention groups were analysed by mixed models linear test

^b^Unpaired t test

### Multiplying effect

According to the questionnaire at 6 months, 9% of the students in the intervention group had undertaken the web course HBH once or several times after the lesson in school and 6% had shown it to another person.

## Discussion

Some previous studies report beneficial effects of preparatory activities such as interactive computer sessions, instructional videos or training with avatars in a multiplayer virtual world [8, 9, 26]. In the present study, the interactive web course did not increase the students’ acquisition of practical CPR skills. This is in accordance with Perkins et al. [27] who also found that the use of preparatory e-learning simulation software by health care providers did not improve their CPR skills. Rehberg et al. [28] compared classroom-based and computer-based CPR training and found that the performance of those in the computer group was not as effective in terms of quality of CPR. Means et al. [5] reported that a meta-analysis of empirical studies on the effectiveness of online learning (not in combination with CPR training) showed varying results for different preparatory computer-based activities. According to a meta-analyses by Hattie [29], the average effect of web-based learning was small, although the variability was huge across studies. A contributing factor may be that web-based learning often ignores important educational foundations such as interaction and timely adapted feedback [29, 30]. It is difficult to compare results from different e-learning studies because of differences in the design and content of courses; with some methods, the participants passively acquire knowledge by watching a movie, whereas other methods engage the participant and require actions to complete the programme. Previous studies have shown that hands-on training is important in terms of acquiring and developing good quality practical CPR skills [8, 12, 13, 31]. Further research is needed to investigate the optimal use of web-based learning as a supplemental learning tool [27], because it increases the flexibility of learning in terms of location and time, is available to participants both before and after practical training and can be cost effective [5] if participants come well prepared to practical training. It is a format that can appeal to young people and can be used for rehearsal [13, 31]. All these factors can be included in the evaluation of effectiveness [30].

According to Bandura [32] and social cognitive theory, an individual’s self-efficacy may affect a person’s performance. Self-reported confidence and lack of CPR skills affect willingness to intervene [33, 34]. There were no significant differences between the groups regarding self-estimated knowledge about performing compressions and ventilation. The web course includes films from lifesaving interventions, as we thought they might affect the students emotionally [35]. However, there was no significant difference between the groups regarding how the students stated they would act in an OHCA situation.

The students who took the web course performed significantly better for the theoretical issues compared with the control group at both measurement points with regard to stroke (54% versus 38% of the total score directly after training), AMI (44% versus 28%) and lifestyle factors (90% versus 75%). The clinical importance of this difference is unclear. It is essential that the public is aware of the typical symptoms associated with AMI and stroke [36, 37]. Early recognition of symptoms and an early call for help are essential to the outcome. Delay in seeking medical care could adversely affect the patient’s prognosis [38, 39]. Public awareness of stroke symptoms in Sweden is low and so too is knowledge regarding back pain, abdominal pain and pain in the right arm as symptoms of AMI [40, 41]. Most students in the control group were familiar with slurred speech and chest pain as symptoms of stroke and AMI, which is similar to previous studies with adults [40, 41]. A contributing factor to the fact that both the intervention and the control groups showed more knowledge about stroke symptoms than AMI symptoms may be the Swedish National Stroke Campaign called AKUT (A for face dropping, K for arm/leg weakness, U for slurred speech, and T for time to call 112), which has been ongoing since autumn 2011.

The students in the present study showed great interest in CPR training; most believed that it is important to learn CPR in schools (98% in both groups) and they felt more confident about taking action compared with before the training (intervention group, 82%; control group, 88%), which is similar to results from previous studies [33, 42, 43].

The study was carried out in schools from all socio-economic areas and included 86% of eligible students, strengthening the generalisability of our findings.

### Study limitations

A few schools had limited access to computers or had technical problems with internet access for all students and the web course was then conducted with the entire class. As a result, there is a risk that some students did not actively participate in the online course and therefore did not learn as much as they would if they had done it by themselves and had independently answered all the questions on the web course. This reflects everyday life in school and how schools apply tuition. It is unclear if this could have attenuated differences between groups and, if so, the generalisability of the study results. It is also unclear if the students’ individual digital literacy affected their participation in the web course and the result [44]. However, the web course has a basic format with clear instructions. Some of the issues in the questionnaire were created for this study and have not been externally validated, but comprehension of the issues was tested before study entry and was found to be satisfactory. The tests were not filmed because several students in a pre-study experienced filming as stressful [24], but all tests were conducted in the same way by one investigator.

## Conclusions

The HBH web course, given to 13-year-old students before CPR training, did not influence practical CPR skills or willingness to act in a cardiac arrest situation, but improved the students’ theoretical knowledge of stroke, AMI and lifestyle factors. Further research is needed to investigate if preparatory education in any format could have the potential to enhance CPR skills and willingness to act.

## Additional files

Additional file 1: Modified version of the Cardiff test. (DOCX 19 kb)

Additional file 2: The questionnaire. (DOCX 19 kb)

Additional file 3: Tables with the results on theoretical knowledge of stroke, AMI and lifestyle factors (DOCX 41 kb)

## Abbreviations

AMI: Acute myocardial infarction
App: Mobile application
CPR: Cardiopulmonary resuscitation
ERC: European Resuscitation Council
HBH: Help-Brain-Heart
NS: Not significant
OHCA: Out-of-hospital cardiac arrest
WHO: World Health Organization
