# Supplementary material for: The effect of a national web course “Help-Brain-Heart” as a supplemental learning tool before CPR training: a cluster randomised trial
Source: Scand J Trauma Resusc Emerg Med. 2017 Sep 12;25:93. doi: 10.1186/s13049-017-0439-0 (PMC5596498; doi:10.1186/s13049-017-0439-0)
Supplement: Supplementary file 1 — Modified version of the Cardiff test. (DOCX 19 kb) [file 13049_2017_439_MOESM1_ESM.docx]

**Additional file 1: the modified Cardiff test.**

The modified version of the Cardiff test, adapted to the ERC guidelines of 2010.[21, 23]. The duration of the practical test was 3 minutes. The optimal conduct was maximum 30 seconds for check responsiveness, check respiration and call for help, followed by 2.5 minutes of CPR. During the CPR, the participants were expected to perform at least 5 cycles of 30 compressions and 2 ventilations (30:2). The rules of assessment were pre-specified as follows:

Check responsiveness by talking

2. Yes, if some form of verbal communication as “are you ok” or “how are you”?

1. No, if no attempt at verbal communication was performed
Method: direct observation and real-time registration in the observation schedule by the test leader.

Check responsiveness by shaking
3. Yes, if the rescuer gently shake the victim shoulders.

2. No, if no attempt to shake the victim shoulders occurred.

1. Potentially dangerous, if the rescuer violently shakes the victim´s shoulders so the head lifted up and down against the ground, which can damage the head or the neck.
Method: direct observation and real-time registration in the observation schedule by the test leader.

Open the airway - chin lift, head tilt.

5. Perfect, if one hand on the forehead, two fingertips on the jawbone (not soft tissue) and gently lifted the chin and bent the head back ie by ERC guidelines.

4 Acceptable/partially correct if several indicators are performed, but not all.

3. Attempted other, if the rescuer tried in other ways than ERC recommendation.

2. Only one element is performed or if the rescuer tries but fails.

1. No, if no attempt to open the airway was performed.
Method: direct observation and real-time registration in the observation schedule by the test leader.

Checks respiration - see, listen, feel

2. Yes, if the rescuer did attempts of breath control, even if not all three actions see, listen and feel were performed and although if the total time of the control was less than 10 seconds.

1. No, if no attempt to check for breathing was performed.
Method: direct observation and real-time registration in the observation schedule by the test leader.

Dials 112

The phone number for the emergency medical dispatchers is 112 in Sweden.
2. Yes, dials 112 within the first minute. A call for help without dialling 112 was not enough, since students were instructed they were alone at the site.

1. No, if no attempt to get help was performed.
Method: direct observation and real-time registration in the observation schedule by the test leader.

Compression/ventilation ratio

4. 30:2 (28-32:2), if the rescuer practical applied compressions and ventilations with the relationship 28-32:2 during the whole test. Participants unable to ventilate the manikin but who attempted a ratio of 28-32:2 were registered as such, as they apparently had learned the skill ratio.

3. Other ratio, if the rescuer applied different ratio of compressions and ventilations than 28-32:2.

2. Compressions only.

1. Ventilations only.

Method: Direct observation and real-time registration in combination with data from Laerdal PC Skill Reporter Systems transferred to the scoring sheet after the test.

Hand-position during compression

Incorrect hand-position was recorded if one compression was in the wrong place, since one wrong compression can cause rib fracture or fracture the xiphoid process of sternum.

4. Correct, if the rescuer place the heel of one hand in the centre of the victim’s chest and with the other hand above.

3. Other wrong, if the rescuer performs chest compressions too high up on the sternum or to the side of the sternum.

2. Too low, if the rescuer performs chest compressions too low on the sternum.

1. Not attempted, if no compressions were performed.

Method: Data from Laerdal PC Skill Reporter Systems was transferred to a scoring sheet after the test.

Average compression depth

The ERC guidelines recommend a compression depth of 50-60 mm,[21]. The PC Skill Reporter system version 2.4 measures up to 60 mm compression depth. To avoid that those who compress >60 mm obtain the highest score, highest score was given for an average compression depth of 50-59 mm. Those who compressed >60 mm received 5 points. We chose to retain the 6-point scale, as in previous studies,[24] even though no one could receive 3 points, which would corresponded to a >65 mm compression depth.

6. 50-59 mm.

5. ≥ 60 mm

4. 35-49 mm

2. 1-34 mm

1. Not attempted, if no compressions were performed.

Method: Data from Laerdal PC Skill Reporter Systems was transferred to a scoring sheet after the test.

Total compression counted

6. 140-190

5. ≥ 191

4. 121-139

3. 81-120

2. 1-80

1. Not attempted, if no compressions were performed.

Method: Data from Laerdal PC Skill Reporter Systems was transferred to a scoring sheet after the test.

Average ventilation volume

5. 500-600 ml

4. 1-499 ml

3. ≥ 601 ml

2. 0 ml, if the rescuer tried to do rescue breaths but failed.

1. Not attempted, if no rescue breaths were performed.

Method: Direct observation and real-time registration if the rescuer tried to do rescue breath. Exact volume, from Laerdal PC Skill Reporter Systems, was transferred to the scoring sheet after the test.

Total ventilation counted
5.8-12
4. 1-7
3. ≥ 13
2. 0, if the rescuer tried to do rescue breaths but failed.
1. Not attempted, if no rescue breaths were performed.

Method: Direct observation and real-time registration if the rescuer tried to do rescue breath. Exact number, from Laerdal PC Skill Reporter Systems, was transferred to the scoring sheet after the test.

Total ”hands-off” time
Total hands-off time was the total time when compressions were not being performed (i.e. also includes time for check responsiveness, check respiration and dial 112).

4. 0-60 s

3. 61-90 s

2. 91-135 s

1. 136-180 s

Method: Data from Laerdal PC Skill Reporter Systems was transferred to a scoring sheet after the test.
