# Supplementary material for: The effect of a national web course “Help-Brain-Heart” as a supplemental learning tool before CPR training: a cluster randomised trial
Source: Scand J Trauma Resusc Emerg Med. 2017 Sep 12;25:93. doi: 10.1186/s13049-017-0439-0 (PMC5596498; doi:10.1186/s13049-017-0439-0)
Supplement: Supplementary file 2 — The questionnaire. (DOCX 19 kb) [file 13049_2017_439_MOESM2_ESM.docx]

**Additional file 2:** **Questionnaire used directly after training and at six months follow-up.**

***Questionnaire directly after training***

1) Have you previously practiced
1a) chest compressions? Yes No
1b) ventilations? Yes No

2) Have you ever been in a situation when someone suffered of
2a) suspected stroke? Yes No Do not know
2b) suspected myocardial infarction? Yes No Do not know
2c) suspected sudden cardiac arrest? Yes No Do not know

3) Do you think it is important to learn
 cardiopulmonary resuscitation in school? Yes No Doo not know

4) Do you think that your skills are sufficient to perform
4a) chest compressions? Yes No Do not know
4b) ventilations? Yes No Do not know

5) Are you more confident now than before the
training to act and start CPR? Yes No Do not know

6) You are at home. How would you act if a friend or relative suffered a sudden cardiac arrest. Tick one answer:
I would not dare or want to intervene
I would give chest compressions only
I would give ventilations only
I would give both compressions and ventilations

6b) Enter the reason that you do not dare or want to doo chest compressions?
Lack of knowledge
Afraid to hurt the person
Afraid of transmitted disease
Other reasons
Do not know

6c) Enter the reason that you do not dare or want to do ventilations?
Lack of knowledge
Afraid to hurt the person
Afraid of transmitted disease
Other reasons
Do not know

7a) You are standing at a bus stop. How would you act if an unknown person suffered a sudden cardiac arrest? Tick one answer:
I would not dare or want to intervene
I would give chest compressions only
I would give ventilations only
I would give both compressions and ventilations

7b) Enter the reason that you do not dare or want to doo chest compressions?
Lack of knowledge
Afraid to hurt the person
I do not want to touch a stranger
Afraid of transmitted disease
Other reasons
Do not know

7c) Enter the reason that you do not dare or want to do ventilations?
Lack of knowledge
Afraid to hurt the person
I do not want to touch a stranger
Afraid of transmitted disease
Other reasons
Do not know

What is correct regarding symptoms of stroke?

8a) Pain in one side of the body Yes No Do not know

8b) Pain in both the left and the right side of the body Yes No Do not know

8c) Weakness in one side of the body Yes No Do not know

8d) Weakness in both sides of the body Yes No Do not know

8e) Onset of symptoms occur slowly Yes No Do not know

8f) Onset of symptoms occur quickly Yes No Do not know

8g) Speech difficulties or slurred speech Yes No Do not know

What is correct regarding symptoms of acute myocardial infarction?

9a) Discomfort/pain in right arm Yes No Do not know

9b) Discomfort/pain in left arm Yes No Do not know

9c) Discomfort/pain in the chest Yes No Do not know

9d) Discomfort/pain in right leg Yes No Do not know

9e) Discomfort/pain in left leg Yes No Do not know

9f) Discomfort/pain in the back Yes No Do not know

9g) Discomfort/pain in the stomach Yes No Do not know

9h) Headache Yes No Do not know

9i) Nausea Yes No Do not know

Good living habits provides protection against stroke and

myocardial infarction. Which living habits are protective? Yes No Do not know

10a) Regular strenuous exercise Yes No Do not know

10b) To smoke Yes No Do not know

10c) To eat fruits and vegetables every day Yes No Do not know

10d) Daily use of the computer Yes No Do not know

10e) To eat fish 2-3 times a week Yes No Do not know

10f) To walk or cycle, so-called everyday exercise Yes No Do not know

11) native language?

***Questionnaire at six months follow up***

1a) Have you done a lifesaving intervention in real life after the CPR training? Yes No

1b) If yes, please describe your lifesaving intervention and the situation: _________________________________

2) Do you think it is important to learn
cardiopulmonary resuscitation in school? Yes No Do not know

3) Do you think that your skills are sufficient to perform
chest compressions? Yes No Do not know
ventilations? Yes No Do not know

4) Are you more confident now than before the
training to act and start CPR? Yes No Do not know

5a) You are at home. How would you act if a friend or relative suffered a sudden cardiac arrest. Tick one answer:
I would not dare or want to intervene
I would give chest compressions only
I would give ventilations only
I would give both compressions and ventilations

5b) Enter the reason that you do not dare or want to doo chest compressions?
Lack of knowledge
Afraid to hurt the person
Afraid of transmitted disease
Other reasons
Do not know

5c) Enter the reason that you do not dare or want to do ventilations?
Lack of knowledge
Afraid to hurt the person
Afraid of transmitted disease
Other reasons
Do not know

6a) You are standing at a bus stop. How would you act if an unknown person suffered a sudden cardiac arrest? Tick one answer:
I would not dare or want to intervene
I would give chest compressions only
I would give ventilations only
I would give both compressions and ventilations

6b) Enter the reason that you do not dare or want to doo chest compressions?
Lack of knowledge
Afraid to hurt the person
I do not want to touch a stranger
Afraid of transmitted disease
Other reasons
Do not know

6c) Enter the reason that you do not dare or want to do ventilations?
Lack of knowledge
Afraid to hurt the person
I do not want to touch a stranger
Afraid of transmitted disease
Other reasons
Do not know

What is correct regarding symptoms of stroke?

7a) Pain in one side of the body

7b) Pain in both the left and the right side of the body

7c) Weakness in one side of the body

7d) Weakness in both sides of the body

7e) The onset of symptoms occur slowly

7f) The onset of symptoms occur quickly

7g) Speech difficulties or slurred speech

What is correct regarding symptoms of acute myocardial infarction?

8a) Discomfort/pain in right arm Yes No Do not know

8b) Discomfort/pain in left arm Yes No Do not know

8c) Discomfort/pain in the chest Yes No Do not know

8d) Discomfort/pain in right leg Yes No Do not know

8e) Discomfort/pain in left leg Yes No Do not know

8f) Discomfort/pain in the back Yes No Do not know

8g) Discomfort/pain in the stomach Yes No Do not know

8h) Headache Yes No Do not know

8i) Nausea Yes No Do not know

Good living habits provides protection against stroke and

myocardial infarction. Which living habits are protective? Yes No Do not know

9a) Regular strenuous exercise Yes No Do not know

9b) To smoke Yes No Do not know

9c) To eat fruits and vegetables every day Yes No Do not know

9d) Daily use of the computer Yes No Do not know

9e) To eat fish 2-3 times a week Yes No Do not know

9f) To walk or cycle, so-called everyday exercise Yes No Do not know

10a) How many times have you used/read the app “Save the heart” (including any lesson in school)?
1
2-3
4-5
>5

10b) Have you shown the app for someone else? Yes No Do not know

11a) How many times have you performed the web course Help Brain Heart (including any lesson in school)?
1
2-3
4-5
>5

11b) Have you shown web course Help Brain Heart for someone else?
 Yes No Do not know
