# Supplementary material for: The effect of a national web course “Help-Brain-Heart” as a supplemental learning tool before CPR training: a cluster randomised trial
Source: Scand J Trauma Resusc Emerg Med. 2017 Sep 12;25:93. doi: 10.1186/s13049-017-0439-0 (PMC5596498; doi:10.1186/s13049-017-0439-0)
Supplement: Supplementary file 3 — Tables with the results on theoretical knowledge of stroke, AMI and lifestyle factors (DOCX 41 kb) [file 13049_2017_439_MOESM3_ESM.docx]

**Table S1A.** Theoretical knowledge about stroke directly after training and at 6 months.

|  | CPR training only, directly after  (n=587) | Web course + CPR training, directly after (n=645) | p value | CPR training only, after 6 months (n=549) | Web course + CPR training after 6 months (n=575) | p value |
| --- | --- | --- | --- | --- | --- | --- |
| Stroke - pain one side? |  |  |  |  |  |  |
| - Yes | 202 (34) | 305 (47) | ref | 261 (48) | 323 (56) | ref |
| - No | 84 (14) | 167 (26) | NS | 71 (13) | 85 (15) | NS |
| - Don’t know | 276 (47) | 164 (25) | <0.001 | 213 (39) | 167 (29) | <0.001 |
| - Missing | 25 (4) | 9 (1) |  | 4 (1) | 0 |  |
| Stroke - pain in both sides? |  |  |  |  |  |  |
| - Yes | 97 (16) | 135 (21) | ref | 84 (15) | 78 (14) | ref |
| - No | 165 (28) | 311 (48) | NS | 206 (38) | 271 (47) | NS |
| - Don’t know | 296 (50) | 191 (30) |  | 255 (46) | 226 (39) | NS |
| - Missing | 29 (5) | 8 (1) |  | 4 (1) | 0 |  |
| Stroke - weak in one side? |  |  |  |  |  |  |
| - Yes | 252 (43) | 403 (62) | ref | 260 (47) | 366 (64) | ref |
| - No | 68 (12) | 97 (15) | NS | 62 (11) | 48 (8) | <0.001 |
| - Don’t know | 245 (42) | 138 (21) | <0.001 | 223 (40) | 161 (28) | <0.001 |
| - Missing | 22 (4) | 7 (1) |  | 4 (1) | 0 |  |
| Stroke - weak in both sides? |  |  |  |  |  |  |
| - Yes | 133 (23) | 154 (24) | ref | 114 (21) | 97 (17) | ref |
| - No | 140 (24) | 294 (46) | 0.007 | 183 (33) | 249 (43) | 0.025 |
| - Don’t know | 287 (49) | 188 (29) | 0.003 | 248 (45) | 229 (40) | NS |
| - Missing | 27 (5) | 9 (1) |  | 4 (1) | 0 |  |
| Stroke – symptoms slowly? |  |  |  |  |  |  |
| - Yes | 72 (12) | 141 (22) | ref | 88 (16) | 96 (17) | ref |
| - No | 221 (38) | 318 (49) | NS | 251 (46) | 289 (50) | NS |
| - Don’t know | 260 (44) | 177 (27) | <0.001 | 206 (38) | 190 (33) | NS |
| - Missing | 34 (6) | 9 (1) |  | 4 (1) | 0 |  |
| Stroke – symptoms quickly? |  |  |  |  |  |  |
| - Yes | 320 (55) | 413 (64) | ref | 319 (58) | 375 (65) | ref |
| - No | 49 (8) | 89 (14) | NS | 54 (10) | 64 (11) | NS |
| - Don’t know | 197 (34) | 135 (21) | 0.001 | 172 (31) | 136 (24) | 0.013 |
| - Missing | 21 (4) | 8 (1) |  | 4 (1) | 0 |  |
| Stroke - slurred speech? |  |  |  |  |  |  |
| - Yes | 394 (67) | 572 (89) | ref | 398 (72) | 494 (86) | ref |
| - No | 15 (3) | 13 (2) | NS | 16 (3) | 14 (2) | NS |
| - Don’t know | 156 (27) | 54 (8) | <0.001 | 131 (24) | 67 (12) | <0.001 |
| - Missing | 22 (4) | 6 (1) |  | 4 (1) | 0 |  |
| Mean total score for stroke | 2.7 (2.0) | 3.8 (1.8) | <0.001 | 2.8 (1.6) | 3.2 (1.4) | <0.001 |

Results are presented as n (%) or mean (SD). Differences in proportions between groups were analysed with regression within Generalized estimation equations. Differences in total score between intervention groups were analysed by mixed models linear test. *P-*values <0.05 were considered statistically significant. NS, not significant. Correct answer is highlighted in green. All numbers are rounded to the nearest integer.

**Table S1B.** Theoretical knowledge about AMI directly after training and at 6 months

|  | CPR training only, directly after  (n=587) | Web course + CPR training, directly after (n=645) | p value | CPR training only, after 6 months (n=549) | Web course + CPR training, after 6 months (n=575) | p value |
| --- | --- | --- | --- | --- | --- | --- |
| AMI- pain in right arm |  |  |  |  |  |  |
| - Yes | 52 (9) | 180 (28) | ref | 80 (15) | 110 (19) | ref |
| - No | 185 (32) | 244 (38) | <0.001 | 179 (33) | 228 (40) | NS |
| - Don’t know | 321 (55) | 213 (33) | <0.001 | 286 (52) | 237 (41) | 0.002 |
| - Missing | 29 (5) | 8 (1) |  | 4 (1) | 0 |  |
| AMI- pain in left arm |  |  |  |  |  |  |
| - Yes | 90 (15) | 202 (31) | ref | 99 (18) | 136 (24) | ref |
| - No | 151 (26) | 220 (34) | 0.029 | 157 (29) | 195 (34) | NS |
| - Don’t know | 317 (54) | 217 (34) | <0.001 | 289 (53) | 244 (42) | 0.003 |
| - Missing | 29 (5) | 6 (1) |  | 4 (1) | 0 |  |
| AMI- chest pain |  |  |  |  |  |  |
| - Yes | 395 (67) | 568 (88) | ref | 408 (74) | 482 (84) | ref |
| - No | 12 (2) | 17 (3) | NS | 15 (3) | 9 (2) | NS |
| - Don’t know | 159 (27) | 56 (9) | 0.001 | 122 (22) | 84 (15) | 0.001 |
| - Missing | 21 (4) | 4 (<1) |  | 4 (1) | 0 |  |
| AMI – pain in right leg |  |  |  |  |  |  |
| - Yes | 29 (5) | 58 (9) | ref | 52 (10) | 40 (7) | ref |
| - No | 201 (34) | 355 (55) | NS | 208 (38) | 266 (46) | 0.027 |
| - Don’t know | 327 (56) | 224 (35) | 0.001 | 285 (52) | 269 (47) | NS |
| - Missing | 30 (5) | 8 (1) |  | 4 (1) | 0 |  |
| AMI – pain in left leg |  |  |  |  |  |  |
| - Yes | 45 (8) | 64 (10) | ref | 56 (10) | 60 (10) | ref |
| - No | 187 (32) | 345 (54) | NS | 203 (37) | 246 (43) | NS |
| - Don’t know | 324 (55) | 229 (36) | 0.001 | 286 (52) | 269 (47) | NS |
| - Missing | 31 (5) | 7 (1) |  | 4 (1) | 0 |  |
| AMI - back pain |  |  |  |  |  |  |
| - Yes | 132 (22) | 205 (32) | ref | 153 (28) | 190 (33) | ref |
| - No | 117 (20) | 206 (32) | NS | 122 (22) | 147 (26) | NS |
| - Don’t know | 312 (53) | 227 (35) | <0.001 | 270 (49) | 238 (41) | 0.021 |
| - Missing | 26 (4) | 7 (1) |  | 5 (1) | 0 |  |
| AMI – abdominal pain |  |  |  |  |  |  |
| - Yes | 116 (20) | 194 (30) | ref | 132 (24) | 143 (25) | ref |
| - No | 118 (20) | 220 (34) | NS | 135 (25) | 174 (30) | NS |
| - Don’t know | 323 (55) | 225 (35) ) | <0.001 | 278 (51) | 258 (45) | NS |
| - Missing | 30 (5) | 6 (1) |  | 4 (1) | 0 |  |
| AMI - headache |  |  |  |  |  |  |
| - Yes | 186 (32) | 198 (31) | ref | 187 (34) | 220 (38) | ref |
| - No | 75 (13) | 219 (34) | <0.001 | 72 (13) | 125 (22) | 0.026 |
| - Don’t know | 299 (51) | 222 (34) | 0.035 | 286 (52) | 230 (40) | 0.014 |
| - Missing | 27 (5) | 6 (1) |  | 4 (1) | 0 |  |
| AMI - nausea |  |  |  |  |  |  |
| - Yes | 247 (42) | 347 (54) | ref | 237 (43) | 282 (49) | ref |
| - No | 45 (8) | 84 (13) | NS | 52 (10) | 89 (16) | 0.030 |
| - Don’t know | 267 (46) | 207 (32) | <0.001 | 256 (47) | 204 (36) | 0.004 |
| - Missing | 28 (5) | 7 (1) |  | 4 (1) | 0 |  |
| Mean total score AMI | 2.5 (2.0) | 4.0 (2.0) | <0.001 | 2.6 (2.0) | 2.9 (1.9) | 0.008 |

Results are presented as n (%) or mean (SD). Differences in proportions between groups were analysed with regression within Generalized estimation equations. Differences in total score between intervention groups were analysed by mixed models linear test. *P-*values <0.05 were considered statistically significant. NS, not significant. Correct answer is highlighted in green. All numbers are rounded to the nearest integer.

**Table S1C.** Theoretical knowledge about lifestyle factors directly after training and at 6 months.

|  | CPR training only, directly after  (n=587) | Web course + CPR training, directly after (n=645) | p value | CPR training only, after 6 months  (n=549) | Web course + CPR training, after 6 months (n=575) | p value |
| --- | --- | --- | --- | --- | --- | --- |
| Regular exercise |  |  |  |  |  |  |
| - Yes | 397 (68) | 559 (87) | ref | 349 (64) | 394 (68) | ref |
| - No | 49 (8) | 37 (6) | 0.007 | 86 (16) | 95 (16) | NS |
| - Don’t know | 107 (18) | 44 (7) | <0.001 | 110 (20) | 86 (15) | 0.042 |
| - Missing | 34 (6) | 5 (1) |  | 4 (1) | 0 |  |
| Smoking |  |  |  |  |  |  |
| - Yes | 25 (4) | 22 (3) | ref | 30 (6) | 22 (4) | ref |
| - No | 478 (81) | 604 (94) | NS | 469 (85) | 522 (91) | NS |
| - Don’t know | 54 (9) | 14 (2) | 0.002 | 46 (8) | 31 (5) | NS |
| - Missing | 30 (5) | 5 (1) |  | 4 (1) | 0 |  |
| Eat fruits or vegetables daily |  |  |  |  |  |  |
| - Yes | 473 (81) | 607 (94) | ref | 456 (83) | 515 (90) | ref |
| - No | 16 (3) | 15 (2) | NS | 27 (5) | 16 (3) | 0.038 |
| - Don’t know | 70 (12) | 18 (3) | <0.001 | 62 (11) | 44 (7) | 0.055 |
| - Missing | 28 (5) | 5 (1) |  | 4 (1) | 0 |  |
| Daily use of computer |  |  |  |  |  |  |
| - Yes | 17 (3) | 18 (3) | ref | 15 (3) | 24 (4) | ref |
| - No | 433 (74) | 564 (87) | NS | 445 (81) | 488 (85) | NS |
| - Don’t know | 105 (18) | 56 (9) | NS | 85 (16) | 63 (11) | 0.039 |
| - Missing | 32 (6) | 7 (1) |  | 4 (1) | 0 |  |
| Eating fish 2–3 times/week |  |  |  |  |  |  |
| - Yes | 383 (65) | 577 (90) | ref | 406 (74) | 479 (83) | ref |
| - No | 39 (7) | 22 (3) | <0.001 | 31 (6) | 29 (5) | NS |
| - Don’t know | 137 (23) | 40 (6) | <0.001 | 108 (20) | 67 (12) | 0.002 |
| - Missing | 28 (5) | 6 (1) |  | 4 (1) | 0 |  |
| Everyday exercise |  |  |  |  |  |  |
| - Yes | 476 (81) | 599 (93) | ref | 466 (85) | 525 (91) | ref |
| - No | 17 (3) | 18 (3) | NS | 15 (3) | 12 (2) | NS |
| - Don’t know | 70 (12) | 24 (4) | <0.001 | 64 (12) | 38 (7) | 0.016 |
| - Missing | 24 (4) | 4 (1) |  | 4 (1) | 0 |  |
| Mean total score LF | 4.5 (2.0) | 5.4 (1.2) | <0.001 | 3.2 (1.2) | 3.4 (1.0) | <0.001 |

Results are presented as n (%) or mean (SD). Differences in proportions between groups were analysed with regression within Generalized estimation equations. Differences in total score between intervention groups were analysed by mixed models linear test. *P-*values <0.05 were considered statistically significant. NS, not significant. Correct answer is highlighted in green. All numbers are rounded to the nearest integer.
